# Revealing the Molecular Mechanisms of Ozone-Induced Pulmonary Inflammatory Injury: Integrated Analysis of Metabolomics and Transcriptomics

**DOI:** 10.3390/toxics13040271

**Published:** 2025-04-02

**Authors:** Xiaolei Zhou, Yunnian Guo, Xiaotong Jian, Xinyi Miao, Pengpeng Wang, Xiaoke Wang, Ling Wang, Huaiyong Chen, Feifei Feng

**Affiliations:** 1 Department of Respiratory and Critical Care Medicine, Henan Provincial Chest Hospital, Chest Hospital of Zhengzhou University, Zhengzhou 450003, China; zhoulei1212@163.com; 2 Department of Toxicology, College of Public Health, Zhengzhou University, Zhengzhou 453001, China; ynguo9911@163.com (Y.G.); zzujianxiaotong@163.com (X.J.); xinyimiao2021@163.com (X.M.); 3 Department of Occupational and Environmental Health, College of Public Health, Zhengzhou University, Zhengzhou 453001, China; wp7221@zzu.edu.cn; 4 Department of Occupational Medicine and Environmental Toxicology, School of Public Health, Nantong University, Nantong 226007, China; wxk11628@ntu.edu.cn; 5 Faculty of Medicine, Macau University of Science and Technology, Macau 999078, China; lingwang@must.edu.mo; 6 Tianjin Key Laboratory of Lung Regenerative Medicine, Haihe Hospital, Tianjin University, Tianjin 300072, China

**Keywords:** ozone, metabolomics, transcriptomic, inflammatory damage, glycerophospholipid metabolism

## Abstract

O_3_ (ozone) is an environmental pollutant that can exacerbate inflammatory damage and contribute to respiratory diseases. However, the molecular mechanisms and potential targets for intervention in ozone-induced lung inflammatory injury are not yet known. To address this, our study exposed mice to 0.6 ppm and 1.0 ppm of O_3_ (3 h/d, 14 d), evaluating lung inflammation through histopathological examinations, lung function assessments, and analyses of white blood cells and inflammatory factors in BALF. Furthermore, we employed transcriptomic and non-targeted metabolomic approaches to decipher differentially expressed genes (DEGs) and metabolites in mouse lung tissue from the 1.0 ppm O_3_ exposure group. A comprehensive integration analysis of these omics data was conducted using Pearson correlation analysis. Finally, our findings show that ozone exposure indeed elicits pulmonary inflammation. Transcriptomic analysis identified 311 differentially expressed genes, predominantly implicated in circadian rhythm, IL-17 signaling pathway, and PPAR signaling. Meanwhile, metabolomic profiling revealed 41 differentially regulated metabolites, mainly associated with riboflavin metabolism, glutathione metabolism, and ABC transporter pathways. Integrated multi-omics analysis through Pearson correlation identified three key components (Pla2g10, O-phosphoethanolamine, and phosphorylcholine) showing significant enrichment in glycerophospholipid metabolism. Collectively, our findings suggest that glycerophospholipid metabolism may serve as potential therapeutic targets and diagnostic biomarkers for ozone-induced pulmonary inflammatory injury.

## 1. Introduction

It is well known that air pollution poses a serious threat to human health and ecosystems [1]. O_3_ (ozone), as one of the most important air pollutants, has been on the rise in recent years amid climate change and can induce chronic respiratory inflammatory diseases [2]. Studies have shown that short- and long-term O_3_ exposure increases global total mortality and respiratory mortality, Bronchial inflammation, and airway hyperresponsiveness (AHR) [3,4,5]. At the same time, epidemiology shows that O_3_ is mainly positively correlated with cardiovascular and respiratory diseases and is a risk factor for morbidity [6]. Animal studies indicate that acute O_3_ exposure triggers airway and lung inflammation, oxidative stress, etc., in mice, while chronic exposure also causes lung pathology and dysfunction [7]. However, the molecular mechanism by which ozone causes these changes in the lungs is still unclear and needs to be explored.

In recent years, multi-omics approaches have been used as an important means to study the pathogenesis of diseases. One study used multi-omics profiling to reveal glycerolipid metabolism-associated molecular subtypes and identify ALDH2 as a prognostic biomarker in pancreatic cancer [8]. Multiomics techniques are also used to identify candidate lung function-related plasma proteins to pinpoint drug targets for common pulmonary diseases [9]. Transcriptomics enables us to fully and deeply understand the types, quantities, structures, functions, and regulatory mechanisms of all gene transcription products within cells or tissues under specific physiological or pathological conditions in an organism. For instance, ozone exposure has been shown to induce lung inflammation, oxidative stress, and transcriptional dysregulation, including activation of Nos2 and Arg1 [7]. In another study, acute ozone exposure can also alter the expression of clock genes (Per1, Arntl, Cry1, and Rora) and key pathways, leading to lung inflammation [10]. A transcriptomics study of mice exposed to 1.0 ppm O_3_ found ozone-induced lipid peroxidation linked to PPAR signaling, potentially triggering ferroptosis and lung injury, with reduced expression of related genes (Adipoq, Lpl, etc.) [11]. Despite variations in experimental designs (e.g., exposure duration, dose, and species), transcriptomics remains a cornerstone for mechanistic elucidation, yet the molecular pathways underlying ozone-induced lung injury remain incompletely characterized.

Metabolomics aids in unveiling metabolic patterns and mechanisms within organisms, identifying disease-related metabolic pathways and specific metabolites for scientific diagnosis and prevention. Metabolomics goes beyond merely profiling the metabolites in biological samples to the identification of novel biomarkers of disease diagnosis, treatment, progression, and prognosis [12]. An observational study revealed that both short-term and long-term O_3_ (9.08 ± 4.06 ppb) exposure is linked to glycerophospholipid metabolism, with shorter exposure (12–24 h) positively correlating with PC (phosphatidylcholine) and lysoPC (lyso-phosphatidylcholine) levels, while longer exposure (1–2 weeks) shows a negative trend [13]. Additionally, toxicological studies showed that acute O_3_ exposure (1 ppm O_3_, 3 h/d, 1 d) disrupts long-chain fatty acid metabolism in mouse lungs, which can be mitigated by supplementing the fatty acid [14]. In sub-chronic O_3_ exposure (0.5 ppm O_3_, 6 h/d, 90 d) studies, altered lipidomic profiles were found in the lungs and serum of rats [15]. Regardless of exposure dose or duration, O_3_ induces metabolic disruptions, and the underlying mechanisms of O_3_-induced lung metabolic disturbances remain to be studied.

However, mono-omics has limits: transcriptomics shows gene expression changes but not direct gene–phenotype links. Metabolomics reflects phenotypes but cannot regulate metabolites genetically. At present, combined protein–transcriptomics studies are common, but there is much to explore in pulmonary inflammatory injury using transcriptomics and metabolomics. Therefore, in this study, we analyzed transcriptomics combined with untargeted metabolomics of lung tissues from mice exposed to ozone to investigate the molecular mechanisms and the potential targets, which could provide a clue for the prevention and invention of ozone-induced lung inflammatory injury.

## 2. Materials and Methods

### 2.1. Animals and O_3_ Exposure

C57BL/6 male mice (SPF grade, 6–8 weeks old, weight 19–25 g) were used in this experiment (No110324231107098714). The mice were raised in the School of Public Health of Zhengzhou University. The feeding conditions were a 12 h light/dark cycle with controlled temperature (approximately 25 °C) and relative humidity. Throughout the experiment, they were provided ad libitum access to autoclaved food and sterile water, and bedding materials were replaced regularly. All experimental procedures were approved by the Ethics Committee of Zhengzhou University (No ZZUIRB 2023-182).

Rodents are insensitive to O_3_ toxicity, owing to their complex nasal turbinate, lung morphological differences, and high urate and ascorbate concentrations in the airway surfactant [16,17]. For these reasons, 3 times is accepted practice for extrapolating concentrations between primates and rodents [18]. Ozone concentrations can reach 0.2–0.3 ppm in areas of air pollution [19]. Therefore, 0.6 and 1.0 ppm ozone in rodents are equivalent.

According to the research group’s previous study, O_3_ can exceed the standard for more than 10 days, and a stable peak occurs within 3 h in summer. To realistic exposure and dose relationships, 0.6 and 1.0 ppm were selected for 14 days of exposure on a dose and time basis.

The O_3_ exposure system consists of an ozone generator (CH-KTB 2G, Guangzhou Chuanghuan Co., Ltd., Guangzhou, China), a whole-body inhalation system (HOPE-med 8050, Tianjin HOPE Technology Co., Ltd., Tianjin, China), and an ozone monitor (Z-1200, Environmental Sensors Inc., Longwood, FL, USA). In this study, mice were exposed to 0.6, 1.0 ppm ozone for 3 h/d, 14 d. During exposure, ozone concentration was dynamically varied within ±5% of the target concentration via real-time monitoring. The exposure chamber maintained a temperature of 20–25 °C and relative humidity of 40–55%. Mice were exposed after the ozone concentration stabilized.

Thirty mice were randomly divided into three groups: filtered air (FA) control group, 0.6 ppm O_3_ group, and 1.0 ppm O_3_ group. There were 10 mice in each group. Mice in ozone-exposed groups were exposed to 0.6, 1.0 ppm ozone for 3 h/d, 14 d, respectively, and mice in the FA group were exposed to clean filtered air for 3 h/d, 14 d.

**Figure 1:**
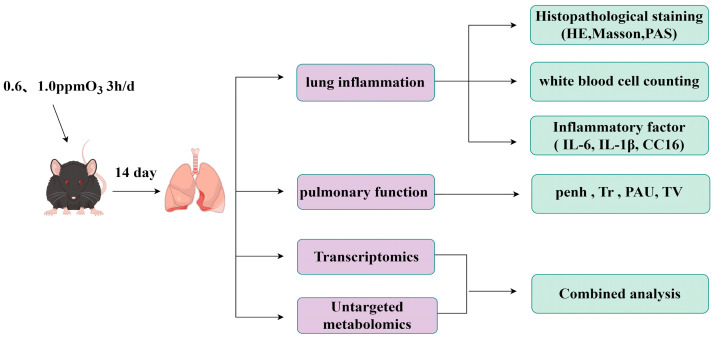
Experimental scheme. The graph is drawn in Figdraw2.0.

After the final exposure, non-invasive pulmonary function tests were conducted on the mice. The mice were anesthetized via intraperitoneal injection with pentobarbital sodium. Subsequently, bronchoalveolar lavage fluid (BALF) and lung tissue were collected and stored at −80 °C. The experimental protocol is illustrated in Figure 1.

### 2.2. Pulmonary Function Testing

Whole body plethysmography (WBP) was used to determine the pulmonary function of mice 24 h after the last exposure. The indices of airway resistance were the airway narrowing index (Penh), relaxation time (Tr), and apnea (PAU). The indicators of early pulmonary fibrosis are tidal volume (TV).

Penh parameter quantifies the level of bronchial constriction. Tr denotes the duration required for a specific volume of air to be exhaled. PAU signifies the temporal disparity between early and late expirations. TV represents the quantity of gas inhaled or exhaled during each breath.

### 2.3. Measurement of Lung Inflammation

#### 2.3.1. Histopathological Staining of Lung

To observe the pathological alterations of the lung, the lung tissue was first fixed in 4% paraformaldehyde fixative solution at room temperature for 24 h. Subsequently, the lung tissue was embedded in paraffin and subjected to a series of processing steps. Following this, the tissue was stained with HE (Hematoxylin and Eosin), Masson’s trichrome, and PAS (Periodic Acid-Schiff) staining to assess inflammatory infiltration, collagen deposition, and goblet cell hyperplasia, respectively.

#### 2.3.2. Collection and Analysis of BALF

BALF was collected for cell counting and inflammatory factor detection. Endotracheal intubation was performed after the mice’s lungs and trachea were exposed after anesthesia. Then, 1 ml of saline was injected to fill the lungs and remained there for a few seconds. The saline is then drained through differential pressure, and BALF (bronchoalveolar lavage fluid) is collected. After centrifugation of BALF, red blood cell lysate (500 μL) was added to the lower sediment for white blood cell counting. (Interleukin-6) IL-6 (Interleukin-1β) and IL-1β were selected as pro-inflammatory markers, while (Clara Cell Secretory Protein 16) CC16 was measured as an anti-inflammatory marker. The supernatant was used to detect the protein levels of IL-6, IL-1β, and CC16 with (enzyme-linked immunosorbent assay) ELISA kits (Wuhan Huamei Biological Engineering Co., Ltd., Wuhan, China).

### 2.4. Transcriptomics

RNA was extracted from mouse lung tissue, with 6 samples from each group. RNA concentration, purity, and integrity were detected. Select a certain number of samples, use the Illumina platform computer sequencing, data volume is 6G/sample. (Sequencing company: Suzhou Panomike Biomedical Technology Co., Ltd., Suzhou, China)

#### Analysis of Differential Expression

DESeq R was used for differential expression analysis, and differentially expressed genes (DEGs) were screened (|log2FoldChange| > 1 and *P*_adjust_ < 0.05). Heatmaps were used to draw clustering heat maps; GO (Gene Ontology) [20] and KEGG (Kyoto Encyclopedia of Genes and Genomes) [21] pathway enrichment analyses were performed to interpret biological functions. 

### 2.5. Untargeted Metabolomics

#### 2.5.1. Sample Extraction

The lung tissues of 6 mice in each group were examined by metabolomics. The lung tissue was taken, and 1.00 mL of methanol and chloroform extract was added, and a steel ball was added; grinding was repeated twice at 50 Hz 60 s. Ultrasound was performed at room temperature for 30 min and then placed on ice for 30 min. Centrifuge for 10 min (12,000 rpm 4 °C), take the supernatant, concentrate, and dry. After that, 200 μL of a 50% acetonitrile solution was added, and the filtrate was transferred to the test bottle for liquid chromatography–mass spectrometry (LC-MS) detection.

#### 2.5.2. Liquid Chromatography Conditions

The LC analysis was performed using the Vanquish UHPLC system (Thermo Fisher Scientific, Waltham, MA, USA). Chromatography was performed on an ACQUITY UPLC^®^HSS T3 (2.1 × 100 mm, 1.8 µm) column (Waters, Milford, MA, USA). The column temperature was maintained at 40 °C. The flow rate was 0.3 mL/min, and the sample size was 2 μL. Then, the positive and negative ion mode operations were carried out, and the specific method is described in detail in this literature [22].

#### 2.5.3. Mass Spectrum Conditions

Mass spectrometric detection of metabolites was performed on Q Exactive Focus (Thermo Fisher Scientific, USA) with an ESI ion source. Simultaneous MS1 and MS/MS (Full MS-ddMS2 mode, data-dependent MS/MS) acquisition was used, specific methods have been introduced [23].

#### 2.5.4. Differential Metabolism Analysis

The conditions for screening differentially expressed metabolites (DEMs) were *P*_adjust_ < 0.05 and VIP > 1, and then clustering and KEGG enrichment analysis were performed. (Sequencing company: Suzhou Panomike Biomedical Technology Co., Ltd., Suzhou, China).

### 2.6. Statistical Analyses

Data were presented as mean ± standard deviation. GraphPad prism 8 software was used for mapping, and SPSS 25.0 statistical software was used for data analysis. One-way ANOVA was used for comparison among multiple groups, LSD test was used to compare the two groups. The correlations among differential genes and metabolites in the lungs were assessed using the Pearson correlation analysis. Statistical significance was set at *p* < 0.05.

## 3. Results

### 3.1. O_3_ Induces Pulmonary Inflammatory Injury in Mice

**Figure 2:**
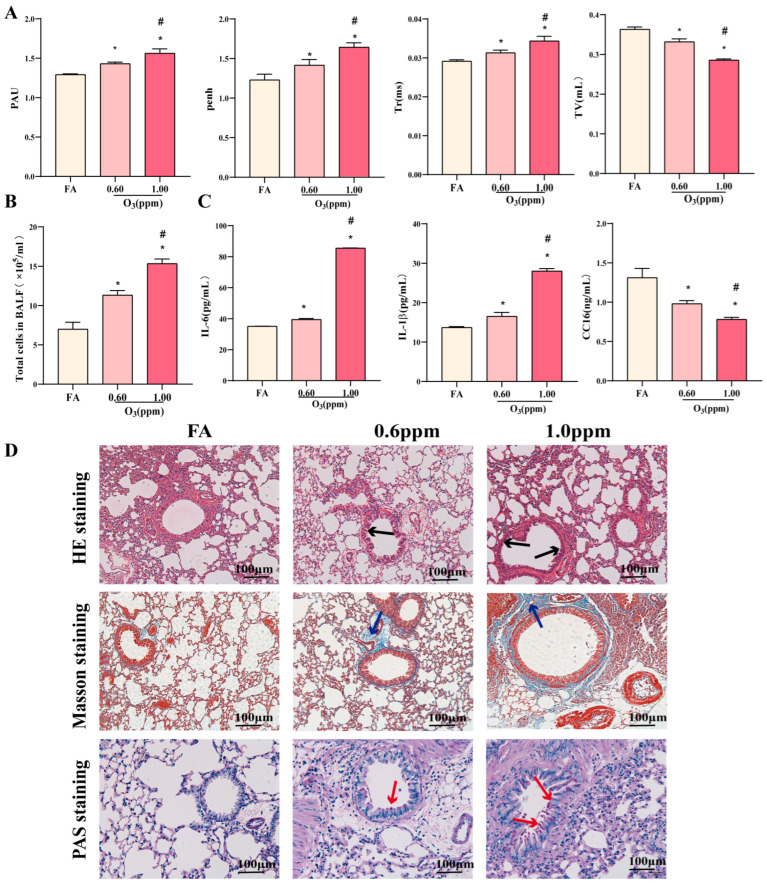
Pulmonary inflammatory injury induced by O_3_. (**A**) Evaluation of lung function, including apnea (PAU), airway narrowing index (Penh), relaxation time (Tr) and tidal volume (TV) (*N* = 10); (**B**) The total cells in BALF (*N* = 10); (**C**) The expression IL-6, IL-1β and CC16 protein in BALF (*N* = 10). (**D**) HE, Masson, PAS staining of lung in mice, the black shows the accumulation of inflammatory cells, Blue staining indicates collagen fiber deposition and the red arrows are the goblet cells (scale bar = 100 μm). Data were shown as mean ± SD, * *p* < 0.05 vs. FA, ^#^ *p* < 0.05 vs. O_3_(0.6 ppm). The graph is drawn from raw data generated by the author.

The change in lung function caused by O_3_ is shown in Figure 2A. Compared to the FA group, the O_3_ group exhibits increased PAU, Penh, and Tr, while TV decreases. Comparing 1.0 ppm to 0.6 ppm O_3_, similar changes suggested that 1.0 ppm caused more severe lung fibrosis and ventilatory issues. In addition, the sO_3_ group showed that upregulation of white blood cells, IL-6, and IL-1β in BALF, with downregulation of CC16. Higher doses caused more severe inflammation (*p* < 0.05) (Figure 2B,C). Histopathological analysis revealed that inflammatory cell infiltration, collagen deposition, and goblet cell hyperplasia were observed in the O_3_ groups, with the O_3_ (1.0 ppm) group exhibiting more severe damage (Figure 2D). Therefore, follow-up experiments used the 1.0 ppm O_3_ group.

### 3.2. Effect of O_3_ Exposure on Transcriptomics of Lung Tissues in Mice

**Figure 3:**
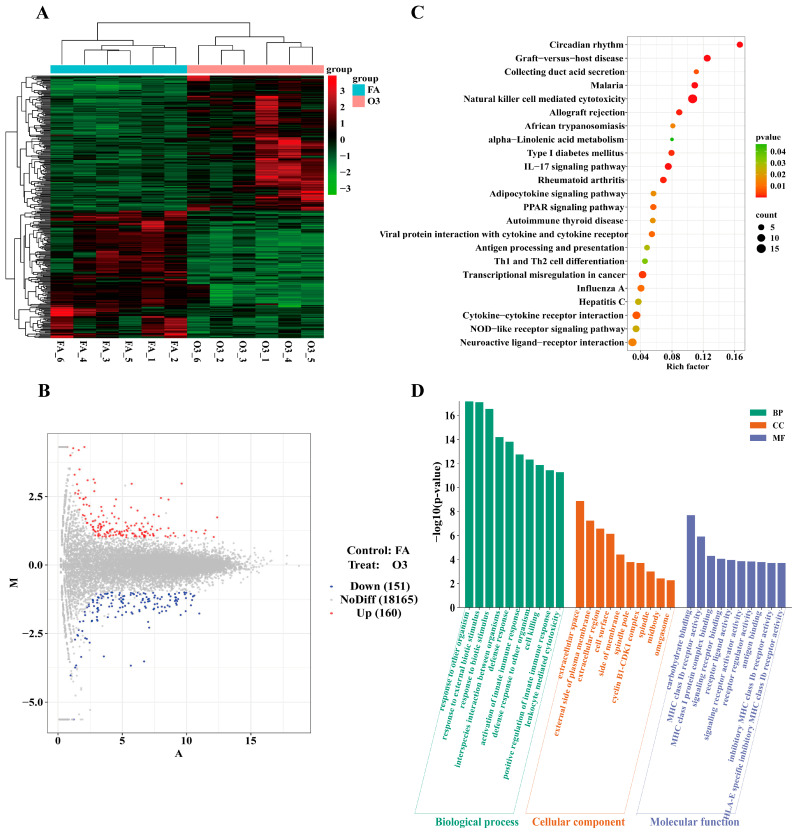
Analysis of differentially expressed genes and pathways. (**A**) Clustering analysis of differentially expressed genes. Red is high expression; green is low expression; (**B**) Differential gene volcano map about O_3_ group vs. FA group. Red dots indicate upregulation, blue dots signify downregulation, and gray dots are unchanged; (**C**) KEGG enrichment analysis on O_3_ group vs. control group; (**D**) GO enrichment analysis of differential genes on O_3_ group vs. FA group. The graph was drawn from the author’s transcriptomic data analysis.

To examine the impact of ozone on lung damage, we conducted transcriptomic profiling of lung tissues from six mice exposed to 1.0 ppm O_3_ versus the FA group. Among 18,476 genes identified, 311 differentially expressed genes (DEGs) were selected based on specific criteria (Table S1). Cluster analysis revealed stark differences between the O_3_ and FA groups (Figure 3A), with 160 genes up-regulated and 151 down-regulated in the O_3_ group. Among them, Pla2g10, Per3, Per2, and Nr1d1 were significantly expressed. (Figure 3B)

To elucidate the biological relevance of these DEGs, we performed KEGG pathway enrichment analysis. The top 20 enriched pathways encompassed immune responses (e.g., natural killer cell-mediated cytotoxicity), circadian rhythm, PPAR signaling, and cytokine signaling (IL-17, cytokine–receptor interaction). Moreover, glycerol phospholipids were enriched. Most of these pathways are related to environmental signal processing, metabolism, and the organism (Figure 3C).

GO enrichment analysis focused on biological processes related to responses to external stimuli, including other organisms and biological agents. Cell component enrichment centered on extracellular spaces and plasma membrane regions, while molecular functions were dominated by receptors and ligands, suggesting pivotal roles in O_3_-induced lung injury mechanisms (Figure 3D).

### 3.3. Effects of O_3_ Exposure on Lung Tissue Metabolomics in Mice

**Figure 4:**
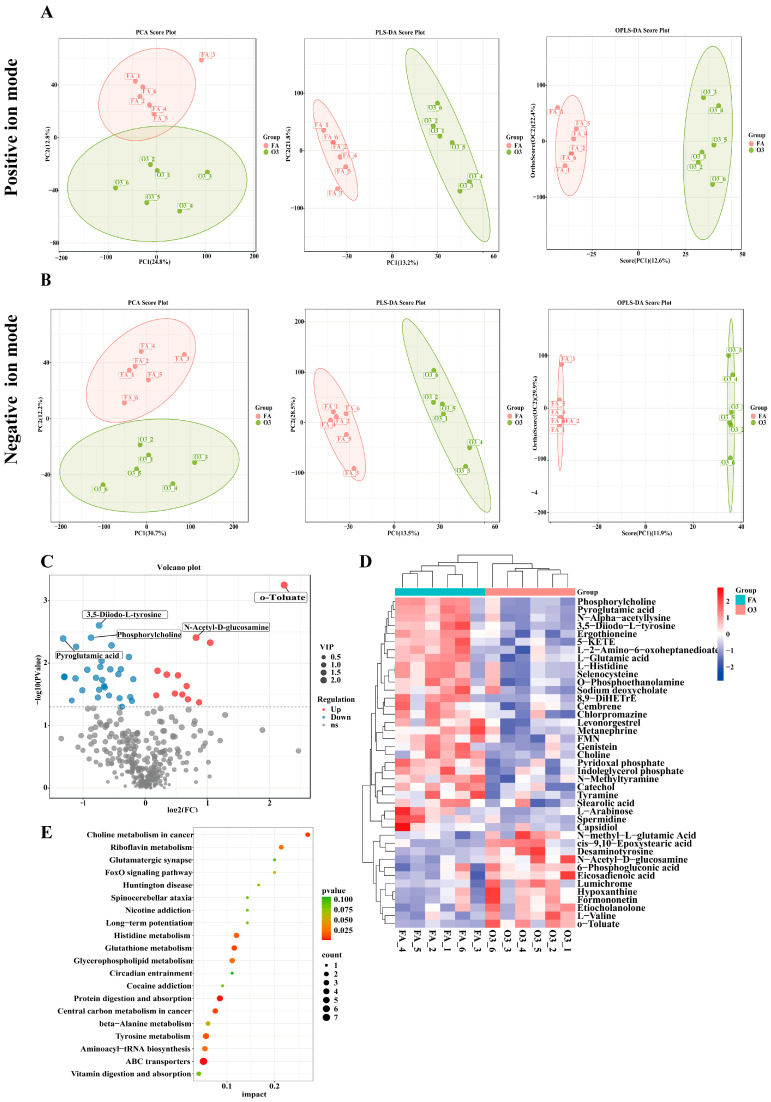
Multivariate statistical analysis of metabolomics analysis of lung tissue. (**A**) PCA, PLS-DA scores, and OPLS-DA scores in positive ion mode; (**B**) PCA, PLS-DA scores, and OPLS-DA scores in negative mode; (**C**) Volcanic map of differential metabolite expression; (**D**) Cluster analysis of differential metabolite expression; (**E**) KEGG enrichment analysis. Red indicates upregulation, blue signifies downregulation, when compared with the FA group. The figure is drawn from the authors’ metabolomics results.

To better understand the damage that ozone causes to the lungs, LC-MC was used to detect the metabolites of lung tissue. Principal component analysis (PCA) showed significant separation among the groups. The Partial Least Squares-Discriminant Analysis (PLS-DA) and Orthogonal PLS-DA (OPLS-DA) scoring plots showed that there were significant differences in metabolic characteristics between the two groups in the positive and negative ion mode (Figure 4A,B).

A total of 354 different metabolites were identified using the Human Metabolome Database (HMDB) [24], MassBank, Lipid Maps, (Advanced Mass Spectral Database) mzCloud™, KEGG, the Nomi Metabolite Standard Database, and other spectrogram databases. According to *P*_adjust_ < 0.05 and VIP _value_ > 1, 41 differential metabolites were identified (Table S2) in comparison with the FA group. In group O_3_, 12 metabolites are up-regulated, and 29 metabolites are down-regulated. o-Toluate, pyridoxal phosphate, eicosadienoic acid, and PC (phosphorylcholine) were significantly expressed (Figure 4C). The clustering analysis of differential metabolites revealed significant differences among the groups, with a notable upregulation of eicosadienoic acid and a significant decrease in L-glutamic acid, O-PEA (O-Phosphoethanolamine), and PC. (Figure 4D).

KEGG enrichment analysis showed that differential metabolites were mainly enriched in the metabolic processes of glycerophospholipid metabolism, ABC transporters, riboflavin metabolism, histidine metabolism, tyrosine metabolism, glutathione metabolism, cancer central carbon metabolism, and so forth (Figure 4E).

### 3.4. Combined Analysis of Transcriptomics and Metabolomics

**Figure 5:**
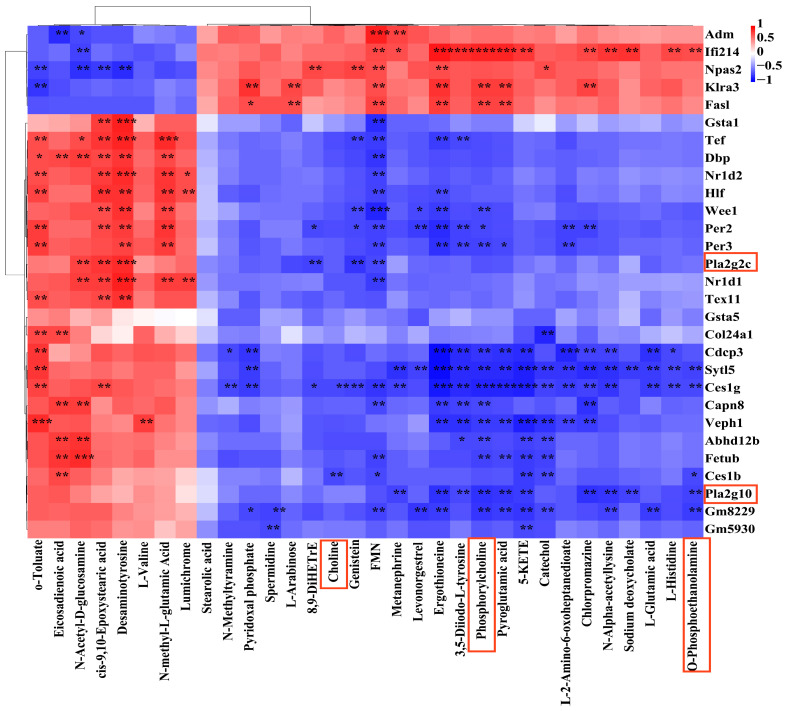
Correlation heatmap between differentially expressed metabolites and genes. The ordinate is the name of different mRNA, and the abscissa is the metabolite. Red represents positive correlation, blue represents negative correlation. The darker the color, the higher the correlation. Asterisks are marked in the heat map to satisfy the results that |r| > 0.7 and *p* < 0.05. (*: *p* < 0.05, **: *p* < 0.01, ***: *p* < 0.001). The graph was developed by the authors based on the results of the joint analysis.

To further clarify the effect of O_3_ on lung injury, the Pearson correlation algorithm of metabolites and genes associated with lung injury was used to draw correlation heatmaps (Figure 5), and the results showed that differential metabolites were associated with differential genes. Among them, Pla2g10 was negatively correlated with O-PEA and PC, but had no correlation with choline (*p* > 0.05). Phospholipase A2 group IIC (Pla2g2c) was negatively correlated with FMN, 8, 9-dihetre, and genistein, and positively correlated with N-acetyl-D-glucosamine, desaminotyrosine, and cis-9,10-epoxystearic acid. Gsta1 was negatively correlated with FMN and positively correlated with desaminotyrosine and cis-9,10-epoxystearic acid.

Then, the association analysis of the two omics was conducted based on the KEGG database, and the common pathway of the two omics was found (Table 1). Combined with the results in Figure 5, we found that the relevant differential genes and differential metabolites were significantly enriched in the glycerophospholipid metabolic pathway. We suspect that the glycerophospholipid metabolic pathway may play an important role in regulating inflammation.

## 4. Discussion

While ozone is a well-established inducer of pulmonary inflammatory injury, the precise molecular mechanisms driving this pathology remain elusive. In this study, the multi-omics method was used to study the mechanism. We found: (1) Ozone does cause pulmonary inflammatory damage (Figure 2), which is consistent with Li’s results [25]; (2) Combined omics showed that glycerophospholipid metabolism may be involved in the occurrence and development of lung inflammation, with O-PEA, PC, and phospholipase A2 Group X (Pla2g10) as potential biomarkers. These findings indicate that ozone may mediate lung inflammation through glycerophospholipid metabolism.

In this experiment, 1.0 ppm O_3_ (3 h/d 14 d) exposure was used for omics analysis of lung tissue. Transcriptomic results indicate that ozone exposure can induce alterations in the circadian rhythm, IL-17 signaling pathway, and PPAR signaling, with genes in these related pathways closely associated with lung inflammation. And the circadian rhythm has the biggest impact; per2, per3, and Nr1d1 are up-regulated, while Npas2 and Arntl (Bmal1) are down-regulated. In past studies, Apagiannakopoulos T pointed out that the imbalance of circadian rhythm can accelerate the development of lung adenocarcinoma [26]. Research has shown that chronic exposure to styrene at concentrations of 20 ppm or higher increases the expression of Per3, Per2, and Nr1d1 in mouse lung tumors while decreasing the expression of Npas2 and Arntl [27]. Similarly, in pigs exposed to ammonia (0.8 ppm), the same changes were observed in the lungs [28]. Despite different methods of exposure, the expression of key genes was consistent with the results. However, Song reported that exposure to air pollution (PM_2.5_) in rats (both pregnant and offspring) led to decreased expression of key clock genes (Per1, Per2, Per3, and Rev-Erbα) and increased Bmal1 expression [29], which contradicts our results. Possible reasons include differences in exposure methods, species, and timing. Furthermore, Nr1d1 overexpression alleviates LPS-induced acute lung injury. Per3 overexpression inhibits NSCLC cell proliferation, induces apoptosis, and suppresses migration and invasion capabilities [30]. Per3 is a pro-cancer gene, and overexpression of Per3 promotes migration and invasion of astroblastoma cells while inhibiting apoptosis of astroblastoma cells and expression of apoptosis genes cleaved-CASP3, P53, and BAX [31]. These findings may contradict our conclusions due to differences in outcomes observed, exposure methods, and roles in different diseases. In summary, circadian rhythms are involved in the occurrence and development of ozone-induced lung inflammation. Future research will delve deeper into its mechanisms.

Metabolomics results revealed significant alterations in glutathione metabolism, ABC transporters, and riboflavin metabolism due to ozone exposure. Among them, glutathione metabolism was most affected, with decreased L-glutamic acid, spermidine, and pyroglutamic acid levels. Studies have shown that preterm infants with a history of bronchopulmonary dysplasia have abnormal airway glutathione metabolism and decreased pyroglutamic acid [32]. L-glutamic acid, essential for GSH synthesis and neurotransmission, is affected by O_3_ exposure. KC noted that O_3_ can remodel glutamatergic synapses. A report indicates that O_3_-induced lung inflammation activates the vagus nerve’s pulmonary afferents and reduces glutamate in the hypothalamus and brainstem in neuropathic pain treatments [32,33]. However, in the study of PCO (Poria cocos oligosaccharides) intervention in LPS (lipopolysaccharide) -induced lung injury, the expression of glutamate in the LPS group is increased [34], which is inconsistent with our conclusions. The possible reasons are different exposure times and exposed substance LPS (0.5 mg/mL, 8 h). Simultaneously, some scholars have pointed out that spermidine can ameliorate endothelial dysfunction and inflammation in mice with pulmonary fibrosis and SLE (systemic lupus erythematosus) [35,36]. Additionally, pyridoxal phosphate, the active form of vitamin B6, has been shown in studies to be a potential candidate drug targeting the SERPINA3 gene in LPS-induced ALI (acute lung injury). It exhibits protective and anti-inflammatory effects in BEAS-2B cells [37]. In summary, glutathione metabolism-related products and pyridoxal phosphate jointly participate in lung inflammation. Further research on their potential mechanisms will provide clues for reducing lung inflammation.

**Figure 6:**
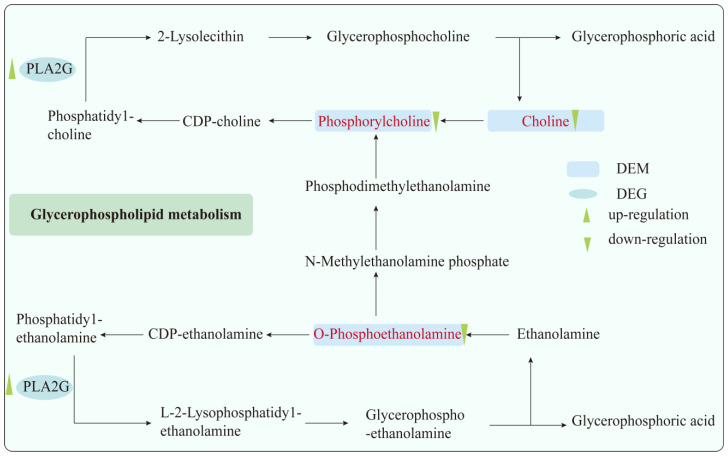
The enriched glycerophospholipid metabolism pathway in this article. Up-regulation and down-regulation indicate that the levels of differentially expressed metabolites (DEMs) or differentially expressed genes (DEGs) were significantly increased or decreased in the 1.0 ppm O_3_ group compared to the FA control group, based on the screening requirements of DEMs and DEGs. The pathway diagram adapted from KEGG with modifications made by the authors.

In this experiment, Pearson correlation analysis and *p*-value were used to quantify the relationship between differential metabolites and differential genes, and the common pathways of the two were jointly analyzed [38]. We found that the glycerophospholipid metabolic pathway had the greatest effect. Studies have shown that ozone exposure is closely related to lipid metabolism, and glycerophospholipid metabolism is associated with acute and chronic ozone exposure [13]. Therefore, we suggest that this pathway may be involved in the occurrence of O_3_-induced pulmonary inflammatory injury. Glycerophospholipid metabolism mainly involves PC and PE (phosphatidylethanolamine) pathways (Figure 6). In the PC pathway, choline is phosphorylated to phosphocholine by CKI1, then reacts with diglyceride to form PC, which is later metabolized into glycerophospholipids and choline (recycled). In the PE pathway, ethanolamine is converted to O-PEA by ETNK, reacts with diglyceride to form PE, and is metabolized into glycerophospholipids and ethanolamine (recycled). PC and PE can interconvert so that they maintain a balance.

O-PEA, a phospholipid crucial for cell membrane construction and metabolism [39], is a precursor of PE. Studies show that Kamikihito, a traditional Chinese medicine, improves psychological and gastrointestinal symptoms in chronic constipation patients, increasing O-PEA levels post-treatment [40]. Probiotic (Q21, Q25, QA85) intervention in Helicobacter pylori-infected patients significantly boosts fecal O-PEA and related metabolic pathways; they can play a role in relieving H. pylori infection [41]. In nerve injuries from explosions or drug abuse, O-PEA levels drop significantly [42,43]. Literature on O-PEA in ozone-induced lung inflammation is limited. One study, however, found that acute 0.8 ppm O_3_ exposure for 3 h increased O-PEA and PC in lipidomics [44], contradicting our hypothesis. Differences may arise from exposure duration, dose, and quantification methods. Studies have shown that the expression of O-PEA in pancreatic tissue is reduced in mice with pancreatitis, and supplementation of this substance can alleviate the severity of cerulein-induced acute pancreatitis [45]. Thus, we hypothesize that O-PEA has anti-inflammatory and anti-anxiety/depression effects in inflammatory and mental disorders.

Lack of choline can lead to pathological changes in the liver, kidneys, and lungs [46]. Phosphocholine, as the carrier and supplement of choline, has been found to reduce inflammatory markers by modulating immune inflammation and inhibiting oxidative stress in asthma patients [47]. In patients with HD (Huntington’s Disease) and R6/2 mice, both choline and phosphocholine levels decrease in the striatum, while glycerophosphocholine increases, suggesting choline metabolism disruption due to GPCPD1 deficiency, which can be ameliorated by citicoline treatment [48]. Respiratory epithelial cells first encounter inhaled ozone, generating limited LOPs from lung phosphocholine. PLA2 stimulates LOPs, increasing IL-6, PGE2, and IL-8 release, causing inflammation [49]. Additionally, phosphocholine is considered a lipid-related small antigen; in animal experiments, boosting anti-phosphocholine antibody levels improved chronic inflammation, such as atherosclerosis [50]. Song proposed phosphocholine as a potential intervention for PM_2.5_ cytotoxicity, as it alleviates energy metabolism disruption and cell death by activating fatty acid oxidation and inhibiting phospho1 [51]. Our study found decreased choline and choline phosphate expression, suggesting ozone exposure causes oxidative membrane damage, reduces membrane proteins, and induces inflammation. Although the underlying mechanisms remain unclear, this could help identify potential biomarkers and provide clues for reducing inflammation.

Pla2g2c and Pla2g10 belong to the PLA2 family, key enzymes in phospholipid catabolism, releasing fatty acids like arachidonic acid, implicated in lung injury and inflammation [52]. Previous studies have shown that Pla2g10 expression increases in ciliated cells and KRT5−/KRT17^+^ cells but decreases in AT2 cells in a bleomycin-induced pulmonary fibrosis mouse model, with symptoms improving after intervention [53]. Elevated mRNA and protein levels of serum exosomal Pla2g10 are associated with aggressive characteristics of NSCLC (non-small cell lung cancer), potentially serving as diagnostic and prognostic biomarkers for NSCLC [54]. Pla2g10 is elevated in the airways of asthmatic patients, while Pla2g10 deficiency attenuates airway hyperreactivity, immune responses, and type 2 cytokine production [55]. Studies have found high expression of Pla2g4c, Pla2g2c, Pla2g2d, and Pla2g5 in a mouse model of ABPA (allergic bronchopulmonary aspergillosis) [56]. However, there is no research on Pla2g2c’s role in inflammation, but we speculate it may act like PLA2. Our study found high Pla2g10 and Pla2g2c expression compared to controls, supporting Pla2g10′s pro-inflammatory role. which is consistent with our findings. Notably, this is the first time we have identified Pla2g10 as a potential biomarker for ozone-induced lung injury, and we will further explore the role of this gene in lung injury and its underlying mechanisms.

There are some limitations in this study. Although the glycerophospholipid metabolism pathway was screened by integrated metabolomic and transcriptomic analyses, the role of the differentially expressed metabolites and genes in the glycerophospholipid metabolism pathway was not validated. More studies will be performed in the future to explore the role and mechanism of glycerophospholipid metabolism pathway in ozone-induced lung inflammation, which will provide novel potential targets for the prevention of ozone-related lung diseases. In summary, through transcriptome and non-targeted metabolome analyses, it was found that glycerophospholipid metabolism is involved in the development of ozone-induced lung inflammation, with O-PEA, phosphorylcholine, and Pla2g10 potentially serving as biomarkers. 

## Figures and Tables

**Table 1:** Pathways for enrichment analysis by transcriptomics combined with untargeted metabolomics.

Pathway	Differential Metabolites	Differential Gene	*p* Value
Protein digestion and absorption	L-Glutamic acid; L-Histidine; L-Valine; Tyramine	Col24a1	0.0024
Glutathione metabolism	L-Glutamic acid; Spermidine; Pyroglutamic acid	Gsta1; Gsta5	0.0109
Glycerophospholipid metabolism	O-Phosphoethanolamine; Phosphorylcholine; Choline;	Pla2g10; Pla2g2c	0.0255

## Data Availability

The original contributions presented in this study are included in the article/Supplementary Material. Further inquiries can be directed to the corresponding authors.

## Supplementary Materials

The following supporting information can be downloaded at: https://www.mdpi.com/article/10.3390/toxics13040271/s1, Table S1: Different differential gene expression; Table S2: Differential express metabolite expression.

## Abbreviations

AHR	Airway hyperresponsiveness
BALF	Bronchoalveolar Lavage Fluid
CC16	Clara Cell Secretory Protein 16
DEGs	Differentially Expressed Genes
DEMs	Differential metabolites
FA	Filtered Air (Control Group)
GO	Gene Ontology
IL-6	Interleukin-6
IL-1β	Interleukin-1β
KEGG	Kyoto Encyclopedia of Genes and Genomes
LC-MS	Liquid Chromatography-Mass Spectrometry
O-PEA	O-Phosphoethanolamine
PAU	Pause
PC	Phosphorylcholine
Penh	Enhanced Pause
Pla2g10	Phospholipase A2 Group X
PLS-DA	Partial Least Squares-Discriminant Analysis
OPLS-DA	Orthogonal PLS-DA
SPF	Specific Pathogen-Free
Tr	Relaxation Time
TV	Tidal Volume
VIP	Variable Importance in Projection
WBP	Whole Body Plethysmography
PPAR	Peroxisome Proliferator-Activated Receptor
Pla2g2c	Phospholipase A2 group IIC
HMDB	Human Metabolome Database
ELISA	Enzyme-linked immunosorbent assay
