# Supplementary material for: Revealing the Molecular Mechanisms of Ozone-Induced Pulmonary Inflammatory Injury: Integrated Analysis of Metabolomics and Transcriptomics
Source: Toxics. 2025 Apr 2;13(4):271. doi: 10.3390/toxics13040271 (PMC12030830; doi:10.3390/toxics13040271)
Supplement: Supplementary file 1 [file toxics-13-00271-s001.zip › Table S1.pdf]

Different differential gene expression

No	Name	*P*val	Log_2FC	Up/Down
1	Ces1g	0.000000000000	2.446035343	up
2	Fetub	0.000000000000	1.516561784	up
3	Dbp	0.000000000002	2.389112527	up
4	Veph1	0.000000000005	1.003399626	up
5	Tef	0.000000000015	1.023235536	up
6	Per3	0.000000000040	1.245207123	up
7	Per2	0.000000000048	1.078799479	up
8	Hlf	0.000000000596	1.051552049	up
9	Abhd12b	0.000000001878	1.51673997	up
10	Gm8229	0.000000003237	1.435998875	up
11	Alb	0.000000008776	2.396456019	up
12	Cdcp3	0.000000011084	1.757519988	up
13	Nr1d2	0.000000018608	1.263556621	up
14	Nr1d1	0.000000027128	1.20789864	up
15	Wee1	0.000000129785	1.024166712	up
16	Sytl5	0.000000222337	1.52179958	up
17	Gm8220	0.000002894267	2.122169195	up
18	Gm5930	0.000002952774	1.04054332	up
19	Tex11	0.000005716953	1.155500289	up
20	P4ha3	0.000031680728	1.240942524	up
21	Mucl3	0.000031957087	1.238237579	up
22	Ces1b	0.000040066843	1.082551889	up
23	Atp6v0a4	0.000096618933	1.340173502	up
24	Apol7a	0.000105283859	1.697476497	up
25	Capn8	0.000125871973	1.197434319	up
26	Zbtb16	0.000143454883	2.97371184	up
27	Kif14	0.000249434085	1.041627976	up
28	Ildr2	0.000367820609	1.087304981	up
29	Krtap17-1	0.000369017087	1.886098253	up
30	Htr4	0.000374204807	1.568371377	up
31	S100a14	0.000402842159	1.778622682	up
32	Psrc1	0.000477658703	1.444637558	up
33	Pla2g10	0.000548806369	3.492440242	up
34	H60c	0.000573078639	1.101546944	up

Different differential gene expression

No	Name	*P*val	Log_2FC	Up/Down
1	Npas2	0.00000000000	-1.59	down
2	Arntl	0.00000000000	-1.31	down
3	Klra3	0.00000000000	-1.52	down
4	Ifi214	0.00000000001	-1.33	down
5	Ifitm6	0.00000000010	-1.22	down
6	Plac8	0.00000000016	-1.15	down
7	Fasl	0.00000000018	-1.73	down
8	Ms4a4c	0.00000000028	-1.40	down
9	Klra7	0.00000000049	-2.08	down
10	Klri2	0.00000000056	-1.62	down
11	Arhgef37	0.00000000163	-1.19	down
12	Ifi209	0.00000000236	-1.11	down
13	Klhl14	0.00000000414	-1.56	down
14	Ly6c2	0.00000000519	-1.82	down
15	Adm	0.00000000595	-1.26	down
16	Klre1	0.00000000807	-1.41	down
17	Klra4	0.00000001163	-2.29	down
18	H2-Q7	0.00000001205	-1.07	down
19	Trim30b	0.00000001328	-1.15	down
20	Klrd1	0.00000001793	-1.06	down
21	Ms4a4b	0.00000002313	-1.01	down
22	Slfn1	0.00000002466	-1.24	down
23	Il2rb	0.00000004183	-1.27	down
24	Samd3	0.00000012651	-1.51	down
25	Klrb1f	0.00000013085	-1.01	down
26	Asgr1	0.00000014031	-1.35	down
27	Prf1	0.00000015913	-1.54	down
28	Klrb1a	0.00000015918	-1.54	down
29	Klrk1	0.00000021936	-1.28	down
30	Klra9	0.00000025005	-1.56	down
31	Gm9733	0.00000025086	-1.07	down
32	Gzma	0.00000026770	-1.69	down
33	Nkg7	0.00000028068	-1.25	down
34	Spon2	0.00000030553	-1.18	down
