# Supplementary material for: Revealing the Molecular Mechanisms of Ozone-Induced Pulmonary Inflammatory Injury: Integrated Analysis of Metabolomics and Transcriptomics
Source: Toxics. 2025 Apr 2;13(4):271. doi: 10.3390/toxics13040271 (PMC12030830; doi:10.3390/toxics13040271)
Supplement: Supplementary file 1 [file toxics-13-00271-s001.zip › Table S2.pdf]

Differential metabolite expression

No	Name	log_2FC	*P* value	up/down
1	o-Toluate	2.24	0.001	up
2	cis-9,10-Epoxystearic acid	1.05	0.005	up
3	6-Phosphogluconic acid	0.87	0.043	up
4	N-Acetyl-D-glucosamine	0.82	0.004	up
5	Lumichrome	0.69	0.038	up
6	Formononetin	0.67	0.023	up
7	Hypoxanthine	0.6	0.032	up
8	Eicosadienoic acid	0.53	0.016	up
9	N-methyl-L-glutamic Acid	0.48	0.031	up
10	Desaminotyrosine	0.35	0.015	up
11	L-Valine	0.2	0.013	up
12	Etiocholanolone	0.18	0.033	up
13	L-Glutamic acid	-0.19	0.018	down
14	Cembrene	-0.21	0.040	down
15	O-Phosphoethanolamine	-0.24	0.035	down
16	N-Methyltyramine	-0.26	0.008	down
17	Stearolic acid	-0.37	0.050	down
18	Indoleglycerol phosphate	-0.39	0.013	down
19	L-Arabinose	-0.41	0.037	down
20	L-2-Amino-6-oxoheptanedioate	-0.43	0.015	down
21	FMN	-0.54	0.005	down
22	Metanephrine	-0.56	0.013	down
23	L-Histidine	-0.58	0.033	down
24	Selenocysteine	-0.65	0.025	down
25	Sodium deoxycholate	-0.66	0.023	down
26	N-Alpha-acetyllysine	-0.69	0.019	down
27	Genistein	-0.7	0.009	down
28	Chlorpromazine	-0.72	0.012	down
29	Levonorgestrel	-0.74	0.027	down
30	3,5-Diiodo-L-tyrosine	-0.74	0.002	down
31	Tyramine	-0.76	0.036	down
32	8,9-DiHETrE	-0.79	0.017	down
33	Phosphorylcholine	-0.87	0.004	down
34	Choline	-0.93	0.013	down
35	Spermidine	-1.01	0.027	down
36	5-KETE	-1.11	0.005	down

No	name	log2FC	P value	up/down
37	Catechol	-1.12	0.018	down
38	Capsidiol	-1.18	0.040	down
39	Ergothioneine	-1.3	0.017	down
40	Pyridoxal phosphate	-1.3	0.016	down
41	Pyroglutamic acid	-1.32	0.004	down
